# Gender differences in illness perceptions and disease management in patients with gout, results from a questionnaire study in Western Sweden

**DOI:** 10.1186/s12891-023-06416-8

**Published:** 2023-04-15

**Authors:** Ulrika Bergsten, Mats Dehlin, Eva Klingberg, Anton J. Landgren, Lennart T. H. Jacobsson

**Affiliations:** 1 Region Halland, Research and development department, Halmstad, Sweden; 2 Department of Rheumatology and Inflammation Research, Institute of Medicine, Sahlgrenska Academy, University of Gothenburg, Box 480, 405 30 Gothenburg, Sweden; 3 Region Västra Götaland, Research and Development Primary Health Care, Södra Bohuslän Gothenburg, Sweden

**Keywords:** Gender, Gout, Illness perception questionnaire, Patient perspectives

## Abstract

**Background:**

Aims were to examine gender differences in patients with gout with regard to a) self-reported gout severity, b) illness perceptions (IP), c) impact on daily activities and Quality of Life (QoL), d) advice from healthcare professionals, e) having changed dietary- or alcohol habits.

**Methods:**

Adult patients with gout identified in primary and secondary care in Sweden between 2015 and 2017 (*n* = 1589) were sent a questionnaire about demographics, gout disease severity, IP (using the Brief Illness Perception Questionnaire, (B-IPQ)) and disease management. T-tests, Chi square tests, ANalysis Of VAriance (ANOVA) and linear regression models were used for gender comparisons.

**Results:**

Eight hundred sixty-eight patients responded to the questionnaire. Women, *n* = 177 (20%), experienced more severe gout symptoms (*p* = 0.011), albeit similar frequencies of flares compared to men. Women experienced modest but significantly worse IP with regard to consequences, identity, concerns and emotional response (*p* < 0.05) as well as daily activities such as sleeping (*p* < 0.001) and walking (*p* = 0.042) and QoL (*p* = 0.004). Despite this and a higher frequency of obesity in women (38 vs 21%, *P* < 0.001) and alcohol consumption in men (*p* < 0.001), obese women had received significantly less advice regarding weight reduction (47 vs 65%, *p* = 0.041) compared to obese men. On the other hand, women reported having acted on dietary advice to a larger degree.

**Conclusions:**

Despite only modestly worse gout severity and perception, women appear to have been given less information regarding self-management than men. These gender differences should be given attention and addressed in clinical care.

**Supplementary Information:**

The online version contains supplementary material available at 10.1186/s12891-023-06416-8.

## Background

Gout is the most common inflammatory arthritis characterized by acute flares of pain and swelling in one or more joints, most commonly in the first metatarsophalangeal [1]. In addition to acute pain, gout is commonly described to affect quality of life (QoL) and have an impact on daily living [2, 3]. Gout is caused by an immunological reaction to urate crystals deposited in joints and surrounding tissues, in general a consequence of longstanding hyperuricemia. The prevalence of gout is about 1–4% in the western world [1], with increasing incidence and prevalence largely due to an ageing population and the obesity epidemic [1, 4]. The prevalence is 3–fourfold higher in men than in women [5]. Gout is associated with an increased occurrence of several comorbidities such as hypertension, diabetes, renal diseases, obesity and heart failure [6]. Current guidelines recommend different regiments to lower serum urate, which include both pharmacological treatment with urate lowering therapy (ULT) and lifestyle changes [7, 8]. Lifestyle changes could include weight loss, dietary changes such as reduced intake of seafood, meat and alcohol [8]. Long-term treatment with ULT is more potent in reducing urate levels and may even “cure” the disease. In spite of this, management of gout is suboptimal everywhere it has been studied [1], including in Sweden [9], with at most half of patients diagnosed with gout receiving ULT. Lifestyle and dietary adjustments are principal components of self-management of gout.

Gout is more common in men and less well studied in women. Several comorbidities, such as osteoarthritis, obesity, renal disease/failure, hypertension and treatment with diuretics [10] have been reported to be less frequent in women compared to men [11] while alcohol consumption has been reported to be lower in women than in men [11]. Some of these differences are likely due to older age at gout diagnosis in women [6]. Furthermore, ULT has been reported as less frequent in women compared to men [12] whereas the impact of gout on QoL has been reported as greater in women [3, 12]. These factors, in addition to others, such as illness perception (IP), can influence the care provided and possibly lead to gender differences in disease management.

IP is defined as an individual’s idea of their illness. Depending on how patients perceive their illness it could directly influence aspects of the disease management [13]. IP can be measured by the brief illness perception questionnaire (B-IPQ) [14] and has been studied in several different diseases, such as heart diseases [15–17], allergic rhinitis [18], asthma [19], rheumatoid arthritis [20] chronic kidney disease [21] and gout [22, 23]. B-IPQ entails 8 dimensions: Consequences, Timeline, Personal control, Treatment control, Identity, Concerns, Understanding and Emotional response. IP in patients with gout have been studied in both primary [24] and secondary care settings [23]. Both studies showed that IP influences adherence to ULT treatment [23, 24]. In addition, poor IP in gout has been associated with increased mortality [25]. Few studies have assessed gender differences in IP in gout. Dalbeth et al., in a small study comprising 142 patients with gout from primary and secondary care, reported that IP did not differ between men and women with gout [23]. There is thus a relative lack of studies reporting on gender difference in disease severity and perception of gout.

The aims of the present study were therefore to examine gender differences in patients with gout with regard to a) self-reported gout severity, b) IP, c) impact on daily activities and QoL, d) advice from healthcare professionals, e) having changed dietary- or alcohol habits.

## Methods

### Patients

In this cross-sectional questionnaire study patients were recruited from a rheumatology clinic and 12 primary care centers in Western Sweden. All Individuals ≥ 18 years of age and with ≥ 1 International Classification of Diseases (ICD-10) diagnosis of gout (ICD-10 codes M10), documented at a health care visit to a physician during a period of two years (Jan 2015 through Feb 2017) were identified and sent a questionnaire.

### The questionnaire

The questionnaire included questions about gout characteristics (disease duration, frequency of gout flares, total number of gout flares, gout severity, use of allopurinol), comorbidities, demographics, occupational status, education, well being, alcohol use and dietary changes made due to gout, functional status and Brief Illness Perception Questionnaire (B-IPQ). Non-responders received a second mailing of the questionnaire and ten percent of the final non-responders were randomly selected and interviewed by telephone. The proportion of women was larger in non-responders compared to responders, but the telephone interviews indicated no major differences with regard to gout symptoms [26].

### Definition of variables

Educational level was dichotomized into Low (≤ 12 years) and High (> 12 years). Disease duration was defined as years from first gout diagnosis. Occupational status was defined as retired or working/unemployed/student. Obesity was defined as BMI ≥ 30.0 kg/m^2^. To assess well being numeric rating scales (NRS) measuring pain, fatigue and global health were used and a higher score indicates more pain, fatigue and worse general health. Alcohol use was measured by how many Swedish standard units (1 unit equals 12 g alcohol) consumed per week. Functional status was assessed by Health Assessment Questionnaire (HAQ) [27]. Dietary changes made due to gout regarded reduced intake of seafood, meat, organ meat and alcohol with a Yes/No answer and having received advice from health care professionals on dietary changes or weight loss (Yes/No). Gout severity was reported in 5 steps from very mild to very severe and then dichotomized into very mild/mild vs moderate/severe/very severe. Impact of the disease was measured with Gout Assessment Questionnaire (GAQ) [28]) with a 5 point likert scale measuring how much the last gout flare affected them, from not at all (1) to very much (5).

### Brief illness perception questionnaire

B-IPQ includes 8 dimensions: *Consequences* (beliefs regarding how illness will impact one’s life), *Timeline* (beliefs regarding chronicity of illness), *Personal control* (beliefs regarding controllability of illness), *Treatment control* (beliefs regarding controllability of treatment), *Identity* (how the illness is defined by symptoms), *Concerns* (beliefs regarding the concerns of the disease), *Understanding* (how much the illness is understood) and *Emotional response* (the impact of the illness on emotions). The scales are scored on an 11-point NRS scale (0–10), with higher scores representing greater perceived psychological burden of illness.

### Ethics

All participants were informed in writing that the reported data would be published and returning the questionnaire was considered informed consent. Ethical approval was granted from the Ethical Review Board of Gothenburg, Sweden (519–16). The study was carried out in accordance with the Declaration of Helsinki. Informed consent was obtained from all subjects and/or their legal guardian(s).

### Statistics

Data are expressed as mean (standard deviation (SD)) for continuous variables and number (percentages) for categorical variables. Continuous variables were compared with students t-test and categorical variables with Chi-square test. For age-adjusted comparisons between genders, ANalysis Of VAriance (ANOVA) and linear regression models were used. A *p*-value < 0.05 was considered statistically significant. SPSS version 27 (SPSS Inc., IBM, Chicago, USA) was used for statistical analysis.

## Results

Of the 1589 individuals with an ICD-10 diagnosis of gout who were mailed a questionnaire, 868 (54.6%) responded. The proportion of men was 80%, mean age was 70 years for men and 75 years for women (Table 1). The mean disease duration was 12 and 7 years for men and women respectively. Women reported higher scores on NRS (pain, fatigue, global) and HAQ. Around 50% of men and women reported use of allopurinol and about one fifth of both men and women reported a gout flare during the last month.

**Table 1:** Characteristics of the study population (*n* = 868)

Characteristics	Men (n = 691, 80%)	Women (n = 177, 20%)	*p*-value
**Age**	70 (12)	75 (12)	< 0.001
**Education ≥ 12 years**	267 (39)	44 (25)	0.154
**Occupational status—**Retired	482 (70)	143 (85)	0.002
**Disease duration in years**	12 (3)	7 (8)	< 0.001
**Time since last gout flare**			0.168
> 1 year	291 (45)	55 (37)	
1–12 months	225 (35)	62 (41)	
Last month	131 (20)	33 (22)	
**Pain NRS (0–10)**	2.7 (2.5)	4.0 (2.8)	< 0.001
**Global NRS (0–10)**	2.8 (2.4	3.8 (2.7)	< 0.001
**Fatigue NRS (0–10)**	3.6 (2.6)	4.4 (2.9)	< 0.001
**HAQ score**	0.28 (0.51)	0.63 (0.69)	< 0.001
**Obesity, BMI ≥ 30.0 kg/m2**	154 (22)	60 (34)	< 0.001
**Self-reported comorbidities**			
Diabetes	157 (23)	40 (23)	0.654
Kidney disease	55 (8)	18 (11)	0.410
Hypertension	443 (64)	118 (67)	0.067
Myocardial infarction	104 (15)	19 (11)	0.311
Stroke	68 (10)	17 (10)	0.736
**Pharmacological treatment**			
ULT—allopurinol	341 (49)	87 (49)	0.963
Diuretics—loop	104 (15)	48 (27)	0.001
Diuretics – thiazide	69 (11)	27 (15)	0.156
**Alcohol use, standard drinks a normal week**			< 0.001
No use	84 (13)	60 (36)	
< 1	116 (17)	59 (34)	
1–4	190 (28)	35 (21)	
5–9	190 (28)	13 (8)	
10–14	63 (9)	1 (1)	
> 15	31 (5)	1 (1)	

Table 1, Characteristics of the study population stratified by sex, n (%) or mean (SD), students t-test and Chi-square test used for statistical analysis, *HAQ* Health assessment questionnaire, *NRS* Numeric rating scale, *ULT* Urate lowering therapy

**Fig. 1:**
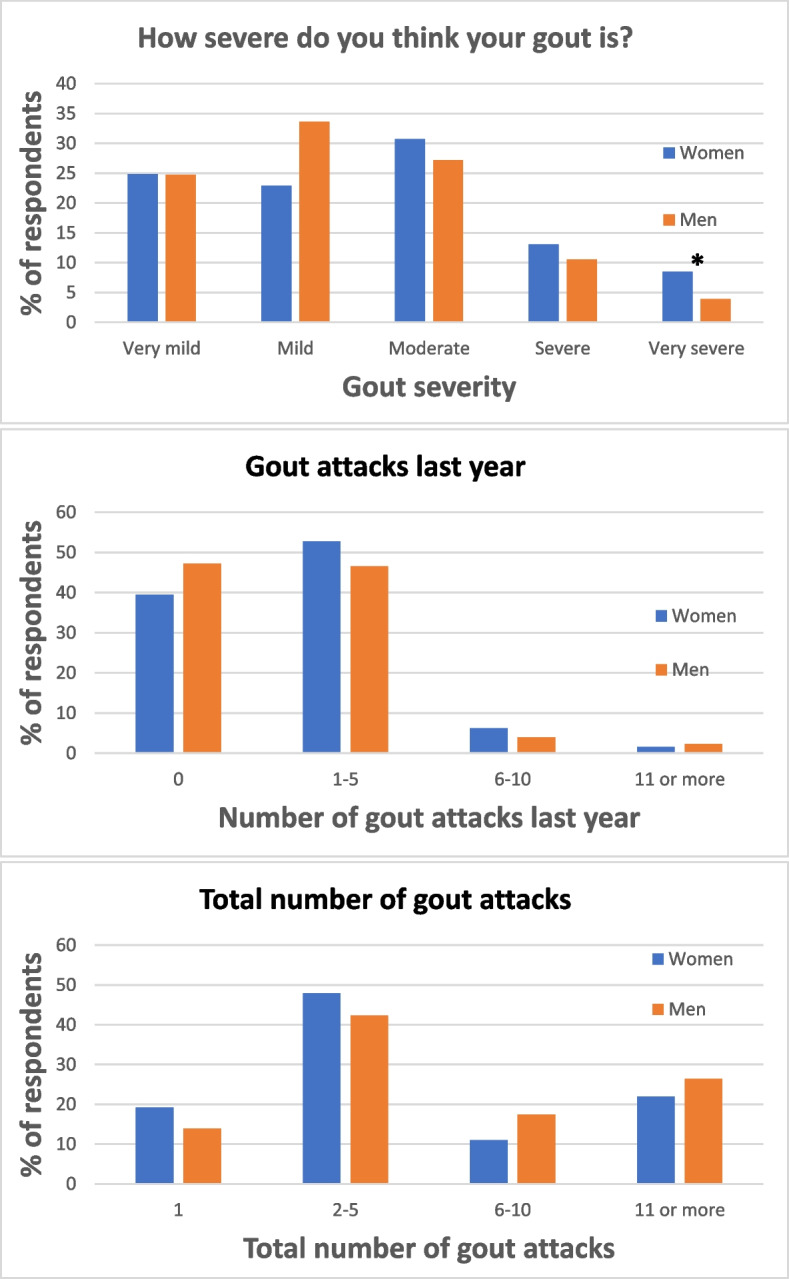
Self-experienced severity of gout and frequency of gout flares stratified by sex. *P*-values are adjusted for age. * = *p* < 0.05

Self-reported severity of gout showed a significant difference between gender, with women stating their gout disease as slightly more severe compared to men also after adjustment for age (Fig. 1). However, in self-reported number of gout flares, both overall and during the last year, there were no differences between genders (Fig. 1).

The B-IPQ also showed slight differences between genders for some of the dimensions (Table 2). Women reported modest but significantly worse IP with regard to consequences, treatment control, identity, concerns and emotional response (*p* < 0.05), differences that remained after adjustment for age. Within the dimensions of personal control, timeline and understanding, there were no significant differences between gender (Table 2). Since men had a significantly longer disease duration we also adjusted for this in addition to age but results were similar with the exception of treatment control that no longer showed a significant difference (data not shown). However, when stratifying men and women into two groups according to self-reported gout severity, all significant differences disappeared with the exception that women with severe gout reported the consequences as worse and women with milder gout were more concerned and emotionally affected (Suppl Table 1).

**Table 2:** Age-adjusted gender comparisons of Brief-Illness Perception Questionnaire

Brief-Illness Perception Questionnaire	Men (*n* = 653)	Women (*n* = 154)	*p*-value
	mean (SD)	mean (SD)	
**1. Consequences:** How much does your illness affect your life? (10 = severely affects life)	2.8 (2.6)	3.8 (3.0)	**< 0.001**
**2. Timeline:** How long do you think your illness will continue? (10 = will continue forever)	6.9 (3.7)	6.8 (3.7)	0.785
**3. Personal control:** How much control do you feel you have over your illness? (10 = extreme amount)	5.8 (3.3)	5.3 (3.5)	0.089
**4. Treatment control:** How much do you think your treatment can help your illness? (10 = extremely helpful)	6.8 (3.0)	6.2 (3.2)	**0.045**
**5. Identity:** How much do you experience symptoms from your illness? (10 = many severe symptoms)	3.3 (2.9)	4.0 (3.1)	**0.011**
**6. Concerns:** How concerned are you about your illness? (10 = extremely concerned)	3.1 (3.0)	3.8 (3.3)	**0.008**
**7. Understanding:** How well do you feel you understand your illness? (10 = very clearly)	5.9 (3.2)	5.4 (3.2)	0.124
**8. Emotional response:** How much does your illness affect you emotionally? (10 = extremely affected)	2.5 (2.8)	3.1 (3.1)	**0.006**

Table 2, Age-adjusted gender comparisons of Brief-Illness Perception Questionnaire, groups compared with logistic regression adjusted for age

**Fig. 2:**
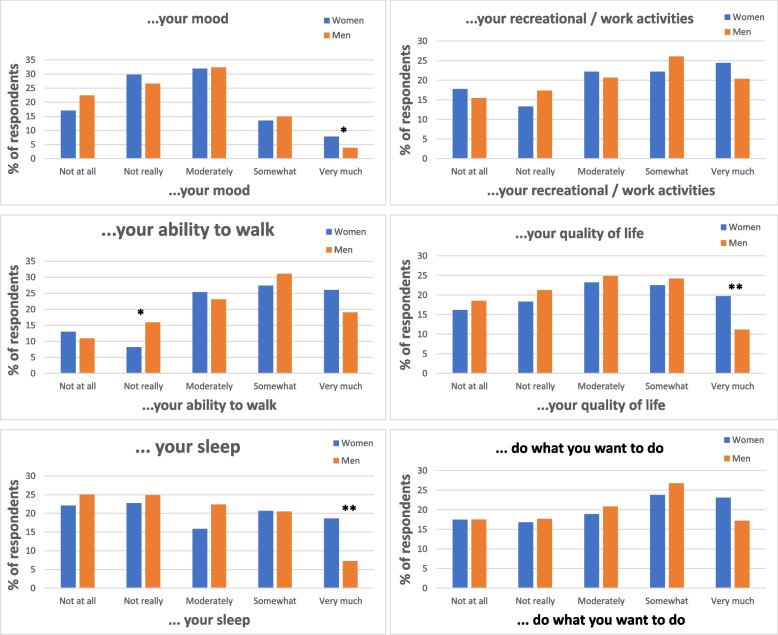
Impact of aspects of daily living during the last gout flare. *P*-values are adjusted for age. * = *p* < 0.05, ** = *p* < 0.005

The impact of gout during last attack, measured by GAQ and adjusted for age, showed significant gender differences in relation to walk, sleep and QoL, with higher impact in women (Fig. 2).

Similar percentages of men and women stated that they had reduced their alcohol consumption (Table 3) but there was a significant gender difference with men reporting a higher alcohol intake (Table 1). Women reported more dietary changes compared to men (Table 3), even though advice from health care personnel regarding dietary changes had more often been given to men (53% vs 22%; *p* < 0.001). Among obese patients (Body Mass Index: BMI ≥ 30 kg/m^2^) more men (65% vs 47%; *p* = 0.041) had been given advice about weight reduction, despite that obesity was considerably less frequent in men (22% vs 34%; *p* < 0.0001, Table 1).

**Table 3:** Age -adjusted gender comparisons of health care advice from health care professionals and dietary changes made by patients in men and women

**Self-reported questions about dietary changes and health care advice**	Men (*n* = 653) **Yes (%)**	Women (*n* = 154) **Yes (%)**	***p*-value**
Have you reduced your intake of seafood?	13	23	**0.001**
Have you reduced your intake of meat?	19	41	**< 0.001**
Have you reduced your intake of organ meats?	45	69	**0.003**
Have you reduced your intake of alcohol?	44	48	**0.018**
Have you been advised by health care professionals about dietary changes?	53	22	**< 0.001**
Have you been advised by health care professionals about weight loss?	31	25	0.385
Have you been advised by health care professionals about weight loss?	65	47	**0.041**

Table 3, Age -adjusted gender comparisons of health care advice from health care professionals and dietary changes made by patients in men and women, groups compared with logistic regression adjusted for age

## Discussion

In this cross-sectional questionnaire study, we explored self-reported experience of gout severity, IP, disease impact, self-care and management in men and women with gout. We found that women reported gout severity, IP, and effects of the disease on daily activities as slightly worse compared to men. Despite these gender differences, women reported having received advice about self-management of gout less frequently than men. On the other hand, women implemented more dietary changes and received allopurinol to the same extent as men.

Self-reported disease severity was slightly worse among women compared to men in this study, which is in line with the study by Harrold et al. 2017 [10]. Whether this experience is a result of the higher frequency of comorbidities in women [6, 10, 11], slight differences in phenotype with different pattern of involved joints [11], a general tendency for women to report worse outcomes in patient-reported outcomes [29] or other factors is unclear.

This study illustrates that IP partly differs between men and women. Gender differences in IP have also been shown for patients with other chronic diseases, such as inflammatory bowel disease [30] and heart disease [17, 31]. Rassart et al. showed that women with congenital heart disease (CHD) experienced more symptoms (identity dimension) and indicated a worse emotional response to the CHD than men [17]. In a study by Marogna et al. 2018 women in cardiac rehabilitation also reported a greater impact of their heart disease on the emotional response dimension in IPQ, compared to men [31]. We show that the self-reported severity of gout was associated with the perceptions of gout (Supplementary Table 1). Similar results were found in a study on heart failure; patients with more severe disease reported worse IP [15]. The consequences of worse IP could be a lower adherence to ULT according to Dalbeth et al. 2011. It is thus important to address and discuss perception of the gout disease regularly with patients [23]. During a gout flare both men and women described that their symptoms interfered with daily activities, but with a higher impact in women with regard to ability to walk, sleep, mood and QoL. Similar results of women having more problems to manage daily activities were also shown in the study by Harrold et al. 2017 [10]. In a study of elderly patients with gout in Spain, Orfila et al. 2006 showed that the difference in QoL between genders could be explained by the fact that women had more chronic conditions and more disability than men [32].

In addition to the patient perspective, the IP of the health care personnel may play a role. In a study of general practitioners (GPs) treating patients with gout, only 4 out of 32 GPs reported that they gave lifestyle advice to patients with gout, despite that many GPs considered dietary factors to be important contributors to gout [33]. Differences between female and male GPs were not examined in that study.

In our study there was a gender difference with regard to self-management strategies and advice from health care professionals. Men and women have been shown to have different educational needs in other chronic rheumatic diseases [34]. In the study by Marques et al. on patients with spondylarthritis there was a significant gender difference, with women stating more educational needs than men with regard to emotional and self-help measures [34]. Gender differences in attitudes regarding management of disease are also in line with our findings that women reported having done more dietary changes. In a systematic review of qualitative studies describing the patient perspective of gout, the main themes were a limited knowledge about gout and the need for long term treatment rather than episodic treatment and the limited knowledge regarding treatment of gout [35]. The impact of gout in relation to gender in a mixed African and African American population from 2014 showed that women were more concerned with joint deformities and footwear, whereas men reported more sexual difficulties [3].

Some limitations should be acknowledged. First, due to the cross-sectional design we were unable to analyze predictors of greater perceived IP or to what extent IP longitudinally affects disease outcome. Second, results may not be applicable for non-responders, although our limited analyses of this group did not demonstrate any major gout phenotype differences. Third, misclassification of gout diagnosis could present a problem, but previous validation studies of gout diagnoses given in our health care system have demonstrated a high validity [36, 37]. In a primary care subset of our study population (consisting of 784 out of the 868 patients with gout), the majority of patients fulfilled the Mexico and Netherlands classification criteria for gout when using ≥ 1 gout diagnosis [37]. For the fourth, we only had data on allopurinol usage (yes/no) but lacked information on doses and effect on urate levels which would have added strength to our study. Finally, the gender differences with regard to advice from health care providers and dietary changes made by patients may not be generalizable to other health care systems.

The study also has several strengths. First, it includes a broad spectrum of patients with a range of disease severity, treated in either primary (the majority of patients) or specialized care. Second, few studies have specifically addressed gender differences in IP, impact on daily life and lifestyle changes made by gout patients.

## Conclusion

In our study we demonstrate that women tend to score their disease as slightly more severe compared to men, although women have an IP similar to men. Despite these overall similarities, in our setting men and women are given different advice regarding disease management and differ with regard to implementation of given advice. These differences call for further investigation of how to tailor and optimize health care advice and treatment of gout in subgroups of patients.

## Supplementary Information

**Additional file 1:** **Suppl Table 1.** Age-adjusted gender comparisons of Brief-Illness Perception Questionnaire stratified by self-reported goutseverity. 

## Data Availability

Data are available upon reasonable request via open or restricted access through a strict controlled access procedure request to the corresponding author.

## Abbreviations

ANOVA: ANalysis Of VAriance
B-IPQ: Brief Illness Perception Questionnaire
GP: General Practitioner
GAQ: Gout Assessment Questionnaire
HAQ: Health Assessment Questionnaire
IP: Illness perceptions
ICD: International Classification of Diseases
NRS: Numeric Rating Scale
QoL: Quality of Life
SD: Standard Deviation
ULT: Urate Lowering Therapy
