# Supplementary material for: Gender differences in illness perceptions and disease management in patients with gout, results from a questionnaire study in Western Sweden
Source: BMC Musculoskelet Disord. 2023 Apr 15;24:300. doi: 10.1186/s12891-023-06416-8 (PMC10105391; doi:10.1186/s12891-023-06416-8)
Supplement: Supplementary file 1 — Additional file 1: Suppl Table 1. Age-adjusted gender comparisons of Brief-Illness Perception Questionnaire stratified by self-reported goutseverity. [file 12891_2023_6416_MOESM1_ESM.docx]

Suppl Table 1, Age-adjusted gender comparisons of Brief-Illness Perception Questionnaire stratified by self-reported gout severity

|  | Gout severity | Men, (n=653), mean (SD) | Women, (n =154), mean (SD) | p-value |
| --- | --- | --- | --- | --- |
| **1. Consequences:** How much does your illness affect your life?  (10=severely affects life) | Mild | 1.6 (1.8) | 1.6 (1.9) | 0.7 |
|  | Severe | 4.5 (2.6) | 5.6 (2.8) | 0.0007 |
| **2. Timeline:** How long do you think your illness will continue?  (10= will continue forever) | Mild | 6.2 (4.0) | 5.5 (4.2) | 0.2 |
|  | Severe | 7.9 (2.9) | 8.0 (2.8) | 0.5 |
| **3. Personal control:** How much control do you feel you have over your illness?  (10=extreme amount) | Mild | 6.4 (3.4) | 5.9 (3.5) | 0.3 |
|  | Severe | 5.0 (3.1) | 4.8 (3.4) | 0.6 |
| **4. Treatment control:** How much do you think your treatment can help your illness?  (10=extremely helpful) | Mild | 7.0 (3.1) | 6.5 (3.2) | 0.3 |
|  | Severe | 6.4 (2.7) | 6.0 (3.0) | 0.3 |
| **5. Identity:** How much do you experience symptoms from your illness?  (10=many severe symptoms) | Mild | 2.1 (2.3 | 2.3 (2.2) | 0.5 |
|  | Severe | 4.9 (2.8) | 5.5 (3.0) | 0.1 |
| **6. Concerns:** How concerned are you about your illness?  (10=extremely concerned) | Mild | 1.8 (2.0) | 2.3 (2.7) | 0.03 |
|  | Severe | 4.9 (3.1) | 5.1 (3.2) | 0.08 |
| **7. Understanding:** How well do you feel you understand your illness?  (10=very clearly) | Mild | 6.1 (3.3) | 5.8 (3.2) | 0.7 |
|  | Severe | 5.7 (3.1) | 5.0 (3.2) | 0.2 |
| **8. Emotional response:** How much does your illness affect you emotionally?  (10=extremely affected) | Mild | 1.3 (1.8) | 1.8 (2.3) | 0.04 |
|  | Severe | 4.0 (3.0) | 4.4 (3.2) | 0.1 |

Suppl Table 1, Age-adjusted gender comparisons of Brief-Illness Perception Questionnaire stratified by self-reported gout severity, groups compared with linear regression adjusted for age
